# A Malignant Gastrointestinal Stromal Tumor of the Gallbladder Immunoreactive for PDGFRA and Negative for CD 117 Antigen (c-KIT)

**DOI:** 10.1155/2011/327192

**Published:** 2011-04-14

**Authors:** Athanasios Petrou, Pari Alexandrou, Alexandros Papalambros, Angelica Saetta, Paraskevi Fragkou, Michalis Kontos, Nicholas Brennan, Antonio Manzelli, Kostantinos Bramis, Evangelos Felekouras

**Affiliations:** ^1^Department of Hepatobilary Surgery, Churchill Hospital, Oxford OX3 7LJ, UK; ^2^Hepatobiliary and Pancreatic Surgical Department, Oxford Radcliffe Hospitals NHS Foundation Trust OXford OX3 9DU, UK; ^3^Department of Pathology, Medical School, University of Athens 11527 Athens, Greece; ^4^First Department of Surgery, Medical School, University of Athens 11527 Athens, Greece

## Abstract

Gastrointestinal stromal tumors (GISTs) compose the largest category of well-recognized nonepithelial neoplasms of the gastrointestinal tract (GI). GISTs of the gallbladder are extremely rare tumors. Only four malignant, two benign and one GIST-like tumor of the gall bladder have ever been described. The four malignant GISTs were all positive for CD 117 antigen (c-kit). We present for the first time a malignant gastrointestinal stromal tumor of the gallbladder, immunoreactive for platelet-derived growth factor receptor alpha (PDGFRA) and negative for CD 117 antigen (c-KIT).

## 1. Introduction

Gastrointestinal stromal tumors (GISTs) are well recognized nonepithelial neoplasms of the gastrointestinal tract (GI). After decades of ultra structural, immunohistochemical, and genetic studies, it is now evident that most GISTs are believed to differentiate from Cajal (ICCs) pacemaker cells. The Cajal pacemaker cells are involved in gastrointestinal tract mobility and regulation of autonomous neuronal transmission [1]. The vast majority of GISTs arises in the stomach (50%). Other common sites include the jejunum and ileum (30%), duodenum (5%), colon and rectum (5%), and the esophagus (5%) [2]. Only seven gallbladder GISTs have ever been reported, and of these, four were malignant [3–9]. These malignant gallbladder GISTs were all positive for CD 117 (c-kit) and CD34 or CD117 alone [4–7]. We report a unique gallbladder GIST which is negative for CD 117 and CD 34 but positive for platelet-derived growth factor receptor alpha (PDGFRA). Apart from the extremely rare site of occurrence, GIST of the gallbladder remains a challenging topic and needs to be examined on the basis of histologic findings, clinical history, and molecular profile with the later having significant impact on the treatment strategy.

## 2. Case Report

A 72-year-old woman was admitted to the First Department of Surgery, University of Athens with symptoms suggestive of obstructive jaundice: fever and chills, dark urine, intermittent right upper quadrant (RUQ) abdominal pain, and jaundice. Clinical examination revealed mild tenderness in the RUQ only with no palpable masses. Initial investigations showed a white cell count (WCC) of 11,556 cells/mm^3^, Total Bilirubin/Direct Bilirubin (TBil/DBil) of 4.80/3.60 mg/dL, alkaline phosphatase (ALP) 440 IU/L, and *γ*-glutamyl transpeptidase (*γ*-GTP) 210 IU/L. Tumor markers were within the normal range. Ultrasound demonstrated a thickened gallbladder wall, bile stones, and moderate common bile duct (CBD) dilatation. Bile duct lithiasis was suspected and an endoscopic retrograde cholangiopancreatography (ERCP) was performed. This demonstrated an almost complete obstruction at the level of the cystic duct and common hepatic duct confluence. However, the underlying pathology could not be identified, and an exploratory laparotomy was decided. This revealed a large irregular hard mass at the site of the gallbladder. The mass was adherent to the duodenum involving the confluence of the cystic duct with the common hepatic duct and the retroduodenal part of the common bile duct. Cholecystectomy was performed along with wedge resection of the gallbladder bed. The common bile duct and common hepatic were excised en bloc with the specimen ensuring free surgical margins along the extrahepatic billiary tree. Lymph node dissection was performed of the hepatoduodenal ligament, the hepatic artery lymph nodes, and the retropancreatic lymph nodes. The enteric-billiary continuity was established through a retrocolic Roux-en-Y hepatico-jejunostomy. The postoperative course was uneventful, and the patient was discharged ten days after operation. 

**Figure 1:**
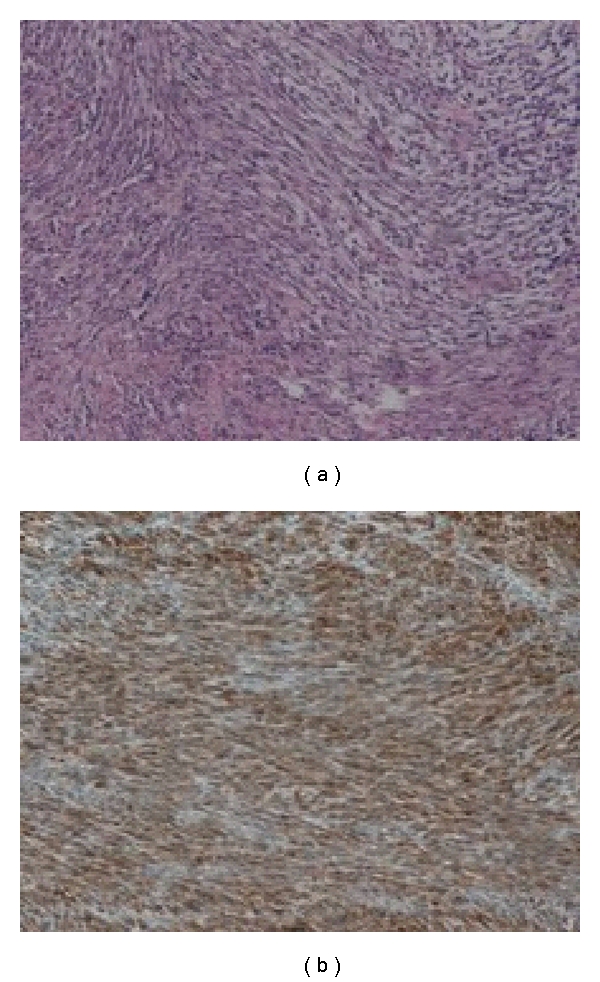
(a) Spindle shaped tumor cells arranged in a short fascicle pattern (HE staining ×200). (b) Tumor cells are diffusely immune-positive for PDGFRA (×200).

In the pathological review, the gallbladder was dilated and measured 7.5 cm × 3.5 cm in size. Within the gallbladder, there was a wide mass covering the entire wall, neck, and body, while the remainder of the mucosal layer was ulcerated. Microscopically, tumor cells infiltrated the muscle layer were spindle shaped in nature, arranged in a short fascicle pattern and immunopositive for PDGFRA (Figures 1(a) and 1(b)). 

The cells demonstrated hypercromatic nuclei, severe pleomorphism and an increased mitotic rate of up to 50 mitosis/50 high power field (HPF). The tumor exhibited focal necrosis and was infiltrating into the pericystic adipose tissue.

## 3. Mutational Analysis of c-Kit and PDGFRA

### 3.1. Material and Methods

**Genomic DNA Isolation**

Ten *μ*m sections were cut from the paraffin-embedded tissue block and treated with xylene/ethanol. The samples were digested overnight at 37°C using proteinase K. DNA was extracted with phenol-chloroform, precipitated in ice-cold ethanol, redissolved in distilled water, and quantitated using a Picodrop Microliter spectrophotometer.

**High-Resolution Melting Analysis**

For the detection of c-Kit exon 9, 11, 13, and 17 mutations as well as PDGFRA mutations in exons 12 and 18, a real-time polymerase chain reaction approach followed by high-resolution melting curve analysis has proved to be a rapid, highly sensitive, and efficient screening method. Subsequently, sequencing of the PCR products is applied for mutation identification. PCR and HRM were consecutively performed on a LightCycler 480 (Roche Diagnostics, GmbH, Germany) in one single run, and all reactions were repeated twice. Each reaction consisted of 20 ng of DNA, 200 nmol/L of each primer, 10 *μ*L of LightCycler LC480 High Resolution Melting Master (Roche), 3.5 mM MgCl_2_ and PCR-grade water adjusted to a total volume of 20 *μ*L.

**Sequencing**

PCR products with an abnormal HRM pattern were sequenced using the Big Dye terminator cycle sequencing kit (Applied Biosystems, California, USA). The sequencing products were analysed on an ABI Prism 310 Genetic Analyzer (Applied Biosystems).

## 4. Results

The specimen was analysed for the presence of activating mutations in exons 9, 11, 13, and 17 of c-Kit gene as well as exons 12 and 18 of PDGFRA gene by performing High Resolution Melting Analysis and Sequencing.

**Figure 2:**
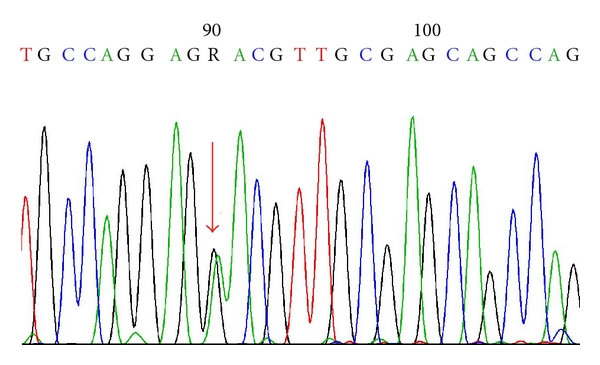
Electropherogram of part of *PDGFRA *sequence. DNA sequencing of silent mutation as c. 2472 C>T, p. V824V.

A mutation at codon 824 coding for valine in exon 18 of PDGFRA gene was detected by high-resolution melting analysis (HRM) and was further confirmed by sequencing as c. 2472 C>T, p. V824V (Figure 2). This silent mutation has previously been described in 2 tumors from the central nervous system (gliosarcomas) as well as in soft-tissue tumors (GISTs).

No c-Kit mutations were found. Results were verified by a second independent PCR reaction and HRM analysis followed by sequencing.

## 5. Discussion

An estimated five-to-six thousand new cases of GIST are diagnosed annually with 10% to 30% of these being malignant [2]. Most tumors are sporadic, affecting individuals in their 5th or 6th decade with some evidence indicating a male predominance [2]. GISTs can also present earlier and this is often seen in one of the rare “GIST syndromes”, which include neurofibromatosis type 1 (NF1), the Carney-Stratakis dyad and familial GIST syndrome [2]. These tumors may occur anywhere in the gastrointestinal tract but most commonly present in the stomach and small intestine. They can also occur in the surrounding structures such as the peritoneum, omentum, liver, pancreas, ovaries, and uterus [2]. Many GISTs are asymptomatic and are discovered incidentally; however, over half of gastric GISTs present with signs of GI bleeding and anaemia with a smaller proportion presenting with abdominal pain or as an abdominal mass [10].

GISTS are diagnosed based on the morphological and immunohistological features. Histology can often be varied, but GISTs are broadly divided into spindle, epithelioid, or mixed cell types. In general, the risk of malignancy is greater in epithelioid tumors than in spindle-celled neoplasms [11, 12]. With the advent of immunohistology, the differentiation of GIST from other mesenchymal tumours has been made possible. Previously, sarcomas, undifferentiated carcinomas, and melanomas would have featured heavily in the differential diagnosis; however, these can largely be excluded with identification or absence of specific immunohistochemical markers [13]. The three most well-described immunohistochemical markers in GISTs are CD117 (c-Kit), PDGFRA, and CD34 [13, 14]. Kit is positive in over 95% of GISTs and only 5–8% of tumors are Kit negative and PDGFRA positive [14]. CD34 is a less sensitve marker for GISTs but is reported in up to 60–70% of the tumors [14]. Miettinen and Lasota who have carried out the largest ever studies on GISTs define these tumors as generally Kit-positive and Kit or PDGFRA mutation-driven mesenchymal tumors of the GI tract with a set of characteristic histologic features including spindle cell, epithelioid, and rarely pleomorphic morphology [14].

We present the case of a malignant GIST of the gallbladder which demonstrated spindle cell morphology and was PDGFRA positive and Kit negative. There have been a small number of gallbladder GISTs described in the literature, of which only four were malignant and all were Kit positive. To our knowledge this presents the first ever recorded presentation of such a GIST. The significance of the Kit negative genotype has implications on the response to further management. The identification of specific cellular markers has led to the development of effective targeted agents, namely, tyrosine kinase inhibitor (TKI) therapy (e.g., imatinib). These have had dramatic effects in prolonging progression free survival in advanced unresectable disease [15]. Most Kit positive tumors are sensitive to imatinib; however, the majority (80%) of PDGFRA mutations are resistant to treatment [16].

Primary GISTs, as in the case here, have the potential for curative treatment with surgical resection. Overall 5-year survival rates for resectable GISTs have been shown to range from 46% to 78.5%; however, predicting the recurrence rate of primary resectable GISTs has been very challenging [17, 18]. The survival rates from the reported gall bladder GISTs are mixed with only short-term followup noted in some of the cases. Although the mutation status is important, the current most important prognostic factor for GISTs is tumor size, mitotic count, and tumor location [19]. This scheme has evolved from studies initially outlined by the National Institute of Health (NIH) and were greatly expanded on by the work of Miettinen and Lasota [13, 14]. The patient in this case had a very large tumor, with 50 mitosis/50 HPF, and was located in the gallbladder. The first two findings alone place this GIST in the high-risk group for recurrence. This is likely compounded by the Kit negative immunohistology which would potentially reduce the benefit of tyrosine kinase inhibitors treatment. It is important to mention that the immunohistochemical examination does not provide information on the exon affected on the type of mutation both of which may be prognostically important.

## 6. Conclusion

This paper, presents a very unique case of GIST, located in the gallbladder, negative for Kit, and positive for PDGFRA. Apart from this being a previously undocumented case, it highlights the challenges in establishing the diagnosis, prognosis, and most effective management for this unpredictable tumour.

## References

[1] Huizinga JD (1999). Gastrointestinal peristalsis: joint action of enteric nerves, smooth muscle, and interstitial cells of Cajal. *Microscopy Research and Technique*.

[2] Liegl-Atzwanger B, Fletcher JA, Fletcher CDM (2010). Gastrointestinal stromal tumors. *Virchows Archiv*.

[3] Ortiz-Hidalgo C, de Leon Bojorge B, Albores-Saavedra J (2000). Stromal tumor of the gallbladder with phenotype of interstitial cells of Cajal: a previously unrecognized neoplasm. *American Journal of Surgical Pathology*.

[4] Mendoza-Marin M, Hoang MP, Albores-Saavedra J (2002). Malignant stromal tumor of the gallbladder with interstitial cells of Cajal phenotype. *Archives of Pathology and Laboratory Medicine*.

[5] Jong KP, Seung HC, Lee S, Ki OM, Sang SY, Hae MJ (2004). Malignant gastrointestinal stromal tumor of the gallbladder. *Journal of Korean Medical Science*.

[6] Peerlinck IDL, Irvin TT, Sarsfield PTL, Harington JM (2004). GIST (Gastro-intestinal Stromal Tumour) of the gallbladder: a case report. *Acta Chirurgica Belgica*.

[7] Furihata M, Fujimori T, Imura J (2005). Malignant stromal tumor, so called "gastrointestinal stromal tumor", with rhabdomyomatous differentiation occurring in the gallbladder. *Pathology Research and Practice*.

[8] Al-Daraji WI, Prescott RJ, Al-Mahmoud RMW, Husain EA, Haider SA (2009). Cytological findings in a primary GIST of the gallbladder. *Cytopathology*.

[9] Al-Daraji WI, Makhlouf HR, Miettinen M (2009). Primary gallbladder sarcoma: a clinicopathologic study of 15 cases, heterogeneous sarcomas with poor outcome, except pediatric botryoid rhabdomyosarcoma. *American Journal of Surgical Pathology*.

[10] Seya T, Tanaka N, Yokoi K, Shinji S, Oaki Y, Tajiri T (2008). Life-threatening bleeding from gastrointestinal stromal tumor of the stomach. *Journal of Nippon Medical School*.

[11] Steigen SE, Eide TJ (2009). Gastrointestinal stromal tumors (GISTs): a review. *APMIS*.

[12] Cichoz-Lach H, Kasztelan-Szczerbińska B, Słomka M (2008). Gastrointestinal stromal tumors: epidemiology, clinical picture, diagnosis, prognosis and treatment. *Polskie Archiwum Medycyny Wewnetrznej*.

[13] Miettinen M, Lasota J (2006). Gastrointestinal stromal tumors: review on morphology, molecular pathology, prognosis, and differential diagnosis. *Archives of Pathology and Laboratory Medicine*.

[14] Lasota J, Miettinen M (2008). Clinical significance of oncogenic KIT and PDGFRA mutations in gastrointestinal stromal tumours. *Histopathology*.

[15] Reichardt P (2010). Optimal use of targeted agents for advanced gastrointestinal stromal tumours. *Oncology*.

[16] Corless CL, Schroeder A, Griffith D (2005). PDGFRA mutations in gastrointestinal stromal tumors: frequency, spectrum and in vitro sensitivity to imatinib. *Journal of Clinical Oncology*.

[17] Naguib SF, Zaghloul AS, El Marakby H (2008). Gastrointestinal stromal tumors (GIST) of the stomach: retrospective experience with surgical resection at the National Cancer Institute. *Journal of the Egyptian National Cancer Institute*.

[18] Cao H, Zhang Y, Wang M (2010). Prognostic analysis of patients with gastrointestinal stromal tumors: a single unit experience with surgical treatment of primary disease. *Chinese Medical Journal*.

[19] Demetri GD, von Mehren M, Antonescu CR (2010). NCCN task force report: update on the management of patients with gastrointestinal stromal tumors. *Journal of the National Comprehensive Cancer Network*.

